# Quality of Reporting of Randomized Clinical Trials in Tai Chi Interventions—A Systematic Review

**DOI:** 10.1093/ecam/nep022

**Published:** 2011-06-23

**Authors:** Jing-Yi Li, Yuan-Fen Zhang, Gordon S. Smith, Chuan-Jiang Xue, Yan-Nan Luo, Wei-Heng Chen, Craig J. Skinner, Joseph Finkelstein

**Affiliations:** ^1^Department of Epidemiology and Preventive Medicine, University of Maryland School of Medicine, Baltimore, USA; ^2^National Study Center for Trauma & EMS, 701 W. Pratt Street, Suite 590, Baltimore, MD 21201, USA; ^3^Department of Kinesiology, University of Maryland, College Park, MD, USA; ^4^Center for Orthopedics, Dongfang Hospital, Beijing, China; ^5^Wangjing Hospital of China Academy of Chinese Medical Sciences, Beijing, China; ^6^Health Outcome Management, LLC, USA; ^7^Welch Center for Prevention, Epidemiology & Clinical Research, Johns Hopkins Medical Institutions, Baltimore, MD, USA

## Abstract

*Objectives*. To evaluate the reporting quality of published randomized clinical trials (RCTs) in the Tai Chi literature following the publication of the CONSORT guidelines in 2001. *Data Sources*. The OVID MEDLINE and PUBMED databases. *Review Methods*. To survey the general characteristics of Tai Chi RCTs in the literature, we included any report if (i) it was an original report of the trial; (ii) its design was RCT; (iii) one of the treatments being tested was Tai Chi; and (iv) it was in English. In addition, we assessed the reporting quality of RCTs that were published between 2002 and 2007, using a modified CONSORT checklist of 40 items. The adequate description of Tai Chi interventions in these trials was examined against a 10-item checklist adapted from previous reviews. *Results*. The search yielded 31 Tai Chi RCTs published from 2002 to 2007 and only 11 for 1992–2001. Among trials published during 2002–2007, the most adequately reported criteria were related to background, participant eligibility and interpretation of the study results. Nonetheless, the most poorly reported items were associated with randomization allocation concealment, implementation of randomization and the definitions of period of recruitment and follow-up. In addition, only 23% of RCTs provided adequate details of Tai Chi intervention used in the trials. *Conclusion*. The findings in this review indicated that the reporting quality of Tai Chi intervention trials is sub-optimal. Substantial improvement is required to meet the CONSORT guidelines and allow assessment of the quality of evidence. We believe that not only investigators, but also journal editors, reviewers and funding agencies need to follow the CONSORT guidelines to improve the standards of research and strengthen the evidence base for Tai Chi and for complementary and alternative medicine.

## 1. Introduction

Randomized controlled trials (RCTs) are generally considered to have the highest level of credibility in determining efficacy of a new treatment, hence, a “gold standard” for evidence-based clinical practice. Many health care professionals make treatment decisions based on reports of RCTs published in peer-reviewed journals.

The randomization process in RCTs not only can control many known and unknown confounding factors, but can also eliminate some bias so that any differences in outcome can be reasonably attributed to the effect of the treatment being tested. Unfortunately, bias can still be introduced into RCTs especially when the trials were poorly conducted. In fact, systematic errors were found in many published RCTs which results in the overestimation of the efficacy of investigational treatments [1, 2]. To identify bias, to assess the validity of a study, and to inform decisions, scientific readers need and deserve to know details regarding the conduct of the trial. Therefore, it is crucial for investigators to specify the design, conduct and analysis in study reports for publication.

Understanding the importance of transparency in reporting clinical trails, an international team, including epidemiologists, statisticians and journal editors, developed the Consolidated Standards for Reporting Trials (CONSORT) statement in 1996 [3]. The statement has a checklist of items that should be included in a trial report along with a flowchart showing the path of trial participants from enrollment to analysis. The goal of the CONSORT statement was to facilitate critical appraisal and interpretation of RCTs by providing investigators a framework for improvement in reporting studies. In addition, journal reviewers can use the CONSORT statement to assist them in identifying reports with potentially biased results. In 2001, the CONSORT statement, the checklist and the flowchart have all been revised in response to pubic feedback. In addition, extensive explanation and elaboration were provided with the revised statement to make it easier for authors and editors to use [4].

Since its first introduction in 1996 and revision in 2001, CONSORT has been used to assess the reporting quality of RCTs testing different interventions, including pharmacology [5], surgery [6], weight loss [7] and acupuncture [8]. Nonetheless, little is known about the quality of reporting in randomized trials of Tai Chi interventions. Therefore, the primary objective of this review was to evaluate the completeness and transparency of Tai Chi trial reports published in peer-reviewed journals. More specifically, we were aiming to (i) describe the characteristics of published Tai Chi RCTs which can be found in MEDLINE between 1966 and 2007; (ii) identify problematic areas in reporting trials among Tai Chi RCTs published between January 2002 and December 2007, using a modified CONSORT checklist of 40 items; and (iii) examine the adequate description of Tai Chi interventions in trials published between January 2002 and December 2007 against a 10-item checklist adapted from previous reviews.

## 2. Methods

### 2.1. Trial Selection

In January 2008, we performed a MEDLINE (1966–2007) search via OVID using key words: *Tai Chi*; *Taiji*; *T'ai Chi*; *randomized controlled trial*; *clinical Trial*. In addition, we latter performed a similar search in PUBMED as suggested by the journal editor. To survey the characteristics of all published trials in the Tai Chi literature, we decided that a trial report would be included if it meets the following criteria: (i) the article was an original report of the trial; (ii) the study design was RCT. For this study, a RCT was defined as an experimental study in which study participants were randomly allocated to at least two groups to test the efficacy/effectiveness of interventions [9]; (iii) one of the treatments being tested was Tai Chi; and (iv) the publication was in English. Three authors, J. L., Y. Z. and J. F., then independently reviewed the abstracts and full articles (when necessary) to assess whether randomized trial reports identified in the literature search met our inclusion criteria. Discrepancies in this assessment were resolved through discussion and consensus. Lastly, among all the included Tai Chi RCTs, we identified those published between January 2002 and December 2007 to examine the quality of reporting of these trials.

### 2.2. Data Extraction

Since the CONSORT statement was not originally designed to be used as a quality assessment instrument, we made the following modification based on the 2001 version of the CONSORT statement.

First, some original items in CONSORT were rewritten so that multiple items were subdivided and listed separately to ensure only one response per item. For instance, item 1 in the original CONSORT statement relating to title and abstract: “How participants were allocated to intervention” was rewritten into two items as the following: (1-1) Does the title identify the report as a RCT? (1-2) Does the abstract have a structured format? This resulted in a checklist including 40 separate items (Table 1). Each item could be assigned a “yes” or “no” to indicate whether the component had been reported as recommended.

Second, item 4 in the original CONSORT statement is described as: “precise details of the interventions intended for each group and how and when they were actually administered”. This item is poorly defined and not readily applied to reporting Tai Chi interventions in a trial. In this review, we used a mini-checklist of 10 items to assess the adequate reporting of testing treatments in a Tai Chi study. If more than seven items in this mini checklist were reported by a trial, item 4 in the original CONSORT statement was considered satisfied. This mini-checklist was adapted from previous reviews [10, 11] and is shown in Table 2. We used this mini-checklist because there is no consensus in the literature in specifying Tai Chi interventions in a RCT. In addition, we believe that this checklist follows the guidelines for reporting non-pharmacologic treatments recommended by the CONSORT group [12].

One author J. L. reviewed full articles of included trials published between 2002 and 2007 to determine whether each of the 40 items in the modified checklist was reported. In addition, a second reviewer, Y. Z., independently checked a set of 10 articles (33% of all studies) selected using computer-generated “random” numbers. Before the evaluation process, J. L. and other authors studied and discussed the definition of each item in the revised CONSORT statement using the published guideline [4]. Furthermore, J. L. played a leading role in rewriting the checklist used in this review. Using the checklist, the authors evaluated five Tai Chi trials that were published before 2002 together, and then completed an independent evaluation of another three ineligible Tai Chi trials due to their publication date. The results were compared and discrepancies were resolved through discussion and consensus. The above process was to ensure that (i) the new checklist was workable and (ii) the single reviewer shared the same interpretation of the checklist with the other authors.

### 2.3. Statistics Analysis

We determined the proportion of RCTs reporting each of the 40 items in the modified CONSORT checklist and each of the 10 items in the mini-checklist. Only descriptive statistics were used. We did not calculate a total quality of reporting score (summing the dichotomized scores of the 40-item checklist) for each trial in this review, as the individual items on the checklist were not felt to share equal weight.

The second rater, Y. Z., reviewed 10 articles and decided whether each of the 40 items in the modified checklist was reported. After both raters finished their review, we calculated Cohen's *κ*-statistics to assess agreement between two reviewers. Good inter-reviewer agreement (*κ* = 0.8–1) was found for the majority (95%) of the CONSORT items. Fair agreement (*κ* = 0.6–0.8) was only found for two items (Item 3-2, which states: “Do authors describe settings where the data were collected?” and item 18, which states: “Do authors report who assigned participants to their groups?”). The disagreements were resolved by consensus between two raters.

## 3. Results

**Figure 1:**
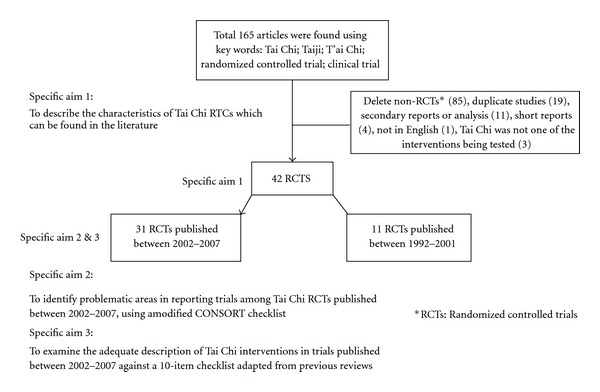
Flow chart of RCTs testing the efficacy of Tai Chi interventions included in this review.

The MEDLINE search yielded total 41 [15–55] relevant Tai Chi randomized trials published from 1992 to 2007. The PUBMED search subsequently identified only one additional study [56]. This article was added to our final analysis. Among the 42 RCTs identified in the literature search, 31 [26–56] of them were published between 2002–2007 and only 11 [15–25] for 1992–2001.  Figure 1 presents a flow chart of studies considered for inclusion.

### 3.1. The Characteristics of Published RCTs Involving Tai Chi between 1966 and 2007

#### 3.1.1. Number of Published Studies

**Figure 2:**
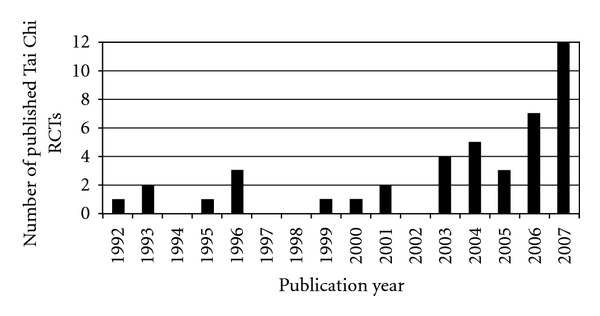
Number of published RCTs testing Tai Chi interventions.

The first report of a RCT of Tai Chi was published in 1992. Since then, the overall number of published studies has been increasing over the years (Figure 2). A dramatic increase in the number of Tai Chi clinical trials is observed in the past 5 years. The number of Tai Chi reports nearly tripled, from only 11 trials in 1992–2001 to 31 trials published between 2002 and 2007.

### 3.2. Distribution of Journals

We found that slightly more than one fourth of the Tai Chi studies (11 trials) were published in the *Journal of the American Geriatrics Society*, while the rest of the trial reports were distributed evenly in 26 other various journals, including *Evidenced Complementary & Alternative Medicine, Age and Ageing, Archives of Physical Medicine & Rehabilitation*, and so forth.

#### 3.2.1. Clinical Application of Tai Chi

We found that in the last two decades Tai Chi has been extensively tested for improving balance and preventing falls among older people. The main study outcome in 16 out of a total of 42 RCTs was associated with balance control, muscle strength and/or number of falls. Nonetheless, researchers were also looking into potential application of Tai Chi in other clinical areas, ranging from anti-hypertension to promotion of general fitness. A list of clinical applications that Tai Chi has been tested for in previous trials is presented in Table 3.

### 3.3. The Reporting Quality of RCTs of Tai Chi Published between 2002 and 2007

A summary of the proportion of RCTs reporting each item in the modified CONSORT checklist is presented in Table 1. In general, the most adequately reported criteria were related to background (item 2), participant eligibility (item 3-1) and interpretation of the study results (item 20-1). In addition, the majority of the reports (90-95%) had well-defined study objectives (item 5) and outcome variables (item 6-1), provided adequate description of statistical methods (item 12), baseline characteristics of study participants (item 15) and a summary of results for study groups (item 17-1). On the other hand, the most poorly reported items were associated with allocation concealment and implementation of randomization (Item 9-1, 9-2, 10-1, 10-2, 10-3). In addition, less than one-fourth (23%) of the trials clearly defined the period of recruitment (item 14-1). Only three trials reported the period of follow-up of their studies (item 14-2). Finally, 23% of included trial reports were considered to provide adequate details of Tai Chi interventions in their studies, because they reported at least 7 out of 10 items in the mini checklist. The extent to which the mini-checklist criteria were met is presented in Tables 2 and 4. More detailedtablescontaining full references are available from the authors.

## 4. Discussion

In order to present their trials in an open and transparent manner, Tai Chi investigators need to follow the widely accepted CONSORT guidelines. It is important to address this issue for many reasons. First, poorly reported clinical trials make it difficult for other researchers to assess the validity of the results, to replicate the study, and to identify gaps that need to be addressed in the design and reporting of future Tai Chi trials [4]. In addition, inadequate reporting may mislead health-care providers in their treatment decisions for patients. Lastly, policy-makers depend on information provided in clinical trials to decide whether they should promote Tai Chi to a larger population.

Unfortunately, despite an increasing number of RCTs assessing Tai Chi in the past two decades, the reporting quality of these trials is sub-optimal and substantial improvement is required to meet the CONSORT guidelines. Almost 50% of the trials we reviewed did not satisfy more than half of the criteria in the modified CONSORT checklist and only 23% of RCTs provided adequate details of Tai Chi intervention used in the trials. Moreover, many of the poorly reported criteria have been associated with biased and erroneous interpretations of study findings. Each of these potentially problematic areas, including randomization, intention-to-treat analysis, masking, sample size and interventions is discussed below to help scientific readers recognize them when reviewing studies of Tai Chi.

### 4.1. Randomization

The results from a RCT are considered the most reliable form of scientific evidence because it involves the random allocation of different treatments to study participants. A well-conducted randomization procedure could not only prevent selection bias, but also control known and unknown confounders, thus ensuring the validity of the study results. Unfortunately, human interference could ruin a perfect randomization method and lead to biased and invalid findings. Therefore, the original CONSORT statement suggests that investigators should provide information regarding who performed randomization and whether allocation sequence was hidden from them, and also how the sequence was generated. This is to help readers assess whether the random assignments were unpredictable, and more importantly, to identify potential bias introduced by the study team. Unfortunately, we found less than half of the trial reports we reviewed provide a description of sequence generation. Reporting of the allocation concealment and randomization implementation was even worse. In more than two-third (70%) of the trials, it was not clear whether the person who allocated interventions knew the sequence or not. This leads a reader to question whether the randomization process was free from human alteration and, in turn, whether the findings were valid.

### 4.2. Intention-to-Treat Analysis

The goal of intention-to-treat analysis is to preserve the benefits of randomization by comparing patients in the treatment groups to which they were originally assigned, not on the treatment eventually administered. The randomization process in a RCT balances potential confounders. Nevertheless, if a confounder is associated with study participation and continuation, then omitting those who withdraw from a trial will reintroduce imbalance on this confounding factor. Thus, the benefit of randomization is compromised if intention-to-treat is not the primary analysis method. However, >60% of the Tai Chi RCTs did not mention the use of intention-to-treat analysis. Among them, 10 trials did not even document the numbers of participants in each group included in each analysis, making it very difficult to judge the validity of their findings.

### 4.3. Masking

The use of masking (blinding investigators/participants to treatment status) is aiming to reduce reporting and measurement bias [57]. In reality, it is not feasible to blind the study participants from the interventions assigned to them in a Tai Chi clinical trial. The investigators, however, could and should make every effort to mask the staff who measure outcomes from the group assignment to eliminate measurement bias. Nonetheless, 17 Tai Chi trials (55%) included in this review did not report any blinding effort. Therefore, it is reasonable to suspect that the results of these studies may be biased intentionally or unintentionally. In fact, it has been shown that, on average, RCTs that have not used appropriate levels of blinding show larger treatment effects than blinded trials [1].

### 4.4. Sample Size

Theoretically, investigators should have a large enough number of subjects in the trials to have a high power of detecting clinical significant difference between interventions, if there is a difference. Ethically, investigators have to have adequate sample size to justify enrolling participants. Unfortunately, more than half (18 studies) of the trials reviewed in the study did not indicate how the sample size was determined. When scientific readers review the results of these trials, they are not able to determine whether the non-significant findings are because Tai Chi was not effective or because the study was underpowered. In addition, even among 13 trials that justified their sample size, five of them did not include attrition in their power calculation. Therefore, these trials were doomed to be underpowered, since the loss of study participants is ubiquitous in RCTs.

### 4.5. Interventions

Ideally, the description of interventions in a clinical trial should provide enough details for other researchers to compare and replicate the treatments. In addition, adequate information regarding the interventions in a trial report could help clinicians to introduce the most effective and efficient treatments into practice [12]. Therefore, it is important for investigators to describe their interventions as clearly as possible. However, when one of the treatments used in a trial is Tai Chi, the investigators have to pay extra attention, mainly because Tai Chi has been recognized as a complex exercise with multiple components. Many of these components are hypothesized to be therapeutic, yet remain poorly understood [11, 58]. Therefore, the Tai Chi interventions emphasizing different components may have different impact on the estimate of the treatment effect [10]. In this review, we used a 10-item mini-checklist to assess the adequate reporting of Tai Chi interventions in the existing clinical trials. We found no trials that met all the criteria; the majority of the trials (35%) reported only 6 out of 10 items in the checklist. Therefore, many of the Tai Chi RCTs could not provide comprehensive details for the interventions being tested. This finding underscores the necessity of developing a guideline for future investigators to report the Tai Chi protocol used in clinical trials.

Our study also revealed other potentially problematic issues when reporting Tai Chi clinical trials. A participant flow chart is the most effective way to help readers to track the numbers of participants in different phases of a complex RCT. Only 48% of the RCTs we reviewed had a diagram showing participant flow. Since the flow charts play an essential role in understanding why some participants did not receive the intervention as assigned, withdrew from the study, or were not counted in the final analysis, the inclusion of the flow charts in reports about Tai Chi interventions is indispensible. In addition, reporting the periods of recruitment and follow-up, including starting and ending dates, was 23% and 10%, respectively in the Tai Chi clinical trials. Providing this information is useful for other researchers to know the rate at which participants were enrolled in Tai Chi studies, and whether a trial was stopped earlier than expected [4]. Lastly, adverse events have not been well documented in the previous Tai Chi trials; only 39% of the reports provided explicit information on adverse events. It is common that, during a clinical trial, study participants may report unintended or undesirable effects. Although these effects may or may not associate with the interventions, readers still deserve to know this information to weigh the risks and benefits of Tai Chi.

### 4.6. Limitations

We acknowledge some potential limitations of this review. First, we did not evaluate the reporting quality of Tai Chi clinical trials published before 2002. Previous studies [4, 57] have shown that the reporting quality of clinical trials for any disease treatment before the 1996 CONSORT statement was extremely poor and that the publication of CONSORT guidelines was associated with improved reporting quality in studies published in journals both using and not using the CONSORT statement [4, 59]. In fact, we found that the average number of items reported in the modified CONSORT checklist is 16.7, 21.8 and 22.0 for trial reports published during 1992–1996, 1997–2001 and 2002–2007, respectively. The poor quality of reporting for Tai Chi trials published between 1992 and 1996 may due to the fact that the CONSORT guidelines were not available to the authors of these trial reports. Therefore, we decided it was only informative to review the Tai Chi trials published after 2002, one year after the publication of the 2001 CONSORT guidelines, since the purpose of our study was to determine how well the Tai Chi RCTs met the new guidelines. Prior to 2002, researchers did not have the strict guidelines for guidance on how to describe their procedures. Second, we only included Tai Chi trial reports found in PUBMED and MEDLINE to ensure that all articles included have undergone similar peer and editorial review ensuring quality control. However, it is important to recognize that Tai Chi studies reported in foreign languages make up a part of Tai Chi literature with an unknown significance. Including foreign language publications may be necessary when the goal of a review is to estimate the effectiveness of Tai Chi interventions. Nevertheless, the primary objective of this article was to evaluate the completeness and transparency of Tai Chi trial reports in the literature and not to evaluate the effectiveness/efficacy of Tai Chi interventions.

Tai Chi trial reports are certainly increasing over the years and play an essential role in determining the efficacy and effectiveness of Tai Chi interventions. While we recognize that the RCTs may not constitute the largest proportion of the Tai Chi literature, they are the “gold standard” by which treatment effectiveness is evaluated. Therefore, we decided to focus this review on reports of randomized trials. Lastly, our two reviewers were not blinded to authorship, journal title and other study-related information because it has been shown that masking has little impact on the conclusions of a systematic review [60, 61]. In addition, it was almost impossible to be totally blind, as this would have meant reformatting all 31 articles included to mask any mention of the author's work.

## 5. Conclusion

As the editors of the *Journal o the American Medical Association*, Drs. Fontanarosa and Lundberg pointed out in their editorial on alternative medicine in 1998 [62], properly designed and conducted randomized controlled trials are needed to provide solid scientific evidence regarding the safety, efficacy and effectiveness of complimentary and alternative medicine (CAM) interventions. As the number of clinical trials testing CAM interventions is increasing, attention needs to be given to the quality of reporting of clinical trials. Without complete and adequate reporting, it is impossible to assess the methodological rigor of the study, and in turn, the scientific evidence of the CAM intervention being tested. We believe that following the CONSORT guidelines will improve the quality of reporting of a RCT. The essential component of CONSORT is transparency of reporting the design, execution and findings of a study, a principle that should apply equally to other types of research. We also believe that not only investigators but also journal editors, reviewers and funding agencies need to follow the CONSORT clinical trial reporting guidelines to improve the standards of research and the strength of the evidence base for Tai Chi and other research.

## Figures and Tables

**Table 1:** Reporting quality of randomized trials testing the efficacy of Tai Chi interventions, using a modified CONSORT checklist, for studies published between 2003 and 2007.

Paper section & topic	Item	Reporting criterion	Number^a^ (%)
Title & Abstract	1-1	Does the title identify the report as a RCT?	20 (65)
	1-2	Does the abstract have a structured format?	27 (87)
Introduction	2	Do authors provide the scientific background and the scientific rationale for their report?	31 (100)
Background			
Methods	3-1	Are eligibility (inclusion and/or exclusion) criteria provided?	31 (100)
Participants	3-2	Do authors describe settings where the data were collected?	9 (29)
Interventions^b^	4	Are the precise details of interventions described?^b^	7 (23)
Objectives	5	Are study objectives or hypotheses specified?	29 (94)
Outcomes	6-1	Are outcome variables clearly defined?	28 (90)
	6-2	Are any particular methods used to enhance the quality of the measurements?	13 (42)
Sample size	7-1	Do authors explain how the sample size was determined?	13 (42)
	7-2	Is attrition taken into account in the sample size calculation?	7 (54)^c^
Randomization: Sequence generation	8-1	Is there a description of the method used to generate the random allocation sequence?	15 (48)
	8-2	Is there any restriction of randomization provided?	14 (45)
Randomization: Allocation concealment	9-1	Is the method used to implement the random allocation sequence described?	5 (16)
	9-2	Do authors clarify whether the sequence was concealed until interventions were assigned?	9 (29)
Randomization: Implementation	10-1	Do authors report who generated the allocation sequence?	8 (26)
	10-2	Do authors report who enrolled participants?	7 (23)
	10-3	Do authors report who assigned participants to their groups?	7 (23)
Blinding (Masking)	11	Do author report whether or not outcome assessors were blinded to group assignment?	14 (45)
Statistical methods	12	Is there a description of the statistical methods used to compare groups for outcome variables?	29 (94)
Results	13-1	Is there a diagram showing participant flow in the trial?	15 (48)
Participant flow	13-2	Do authors report the numbers of participants randomly assigned?	20 (65)
	13-3	Do authors report the numbers of participants receiving intended treatment?	12 (40)
	13-4	Do authors report the numbers of participants completing the study protocol?	23 (74)
	13-5	Do authors report the numbers of participants analyzed for the primary outcome?	16 (52)
Recruitment	14-1	Is the period of recruitment defined including starting and ending dates?	7 (23)
	14-2	Is the period of follow-up defined including starting and ending dates?	3 (10)
Baseline data	15	Are baseline demographic and clinical characteristics of each group presented?	28 (90)
Numbers analyzed	16-1	Do authors report the numbers of participants in each group included in each analysis?	19 (61)
	16-2	Do author state that whether the analysis was by “intention-to-treat”?	12 (39)
	16-3	Are the results presented in absolute numbers?	23 (77)
Outcomes and estimation	17-1	Is there a summary of results for each group?	28 (90)
	17-2	For each outcome variable, is the estimated effect size reported?	26 (84)
	17-3	For each outcome variable, is the effect size's precision (e.g., 95% confidence interval) reported?	24 (77)
Ancillary analyses	18	Do authors report any other analyses performed including subgroup analyses and adjusted analyses, indicating those pre-specified and those exploratory? (e.g., adjusted *P*, *post hoc* or *a posteriori*)	10 (32)
Adverse events	19	Is there any information on adverse events in each intervention group provided?	12 (39)
Discussion	20-1	Do authors address study hypotheses/objectives in their interpretation of the results?	30 (97)
Interpretation	20-2	Do authors describe sources of potential bias or imprecision in their interpretation of the results?	26 (84)
Generalizability	21	Do authors discuss the generalizability (external validity) of the trial findings?	14 (45)
Overall evidence	22	Do authors discuss the results in the context of current evidence?	17 (58)

^a^Number of studies that satisfied reporting criterion.

^b^The adequate reporting of interventions in a Tai Chi clinical trial is assessed against a mini-checklist (Table2). If more than seven items in this mini checklist were reported by a trial, reporting of Tai Chi interventions was considered adequate.

^c^Seven out of thirteen studies included attrition in their sample size justification.

**Table 2:** Assessment of adequate details of Tai Chi interventions, using a mini-checklist adapted from previous reviews, for studies published between 2002 and 2007.

Reporting criterion	Number^a^ (%)
(1) How long was the intervention (weeks)?	31 (100)
(2) Was the Tai Chi training center-based or home-based or both?	28 (90)
(3) If it is center-based, how often was the Tai Chi training class per week and how long did a Tai Chi training class last (minutes)?	28 (90)
(4) What did a Tai Chi training session consist of in the study? Were there any other non-Tai Chi exercises included in a Tai Chi training session?	2 (6)
(5) What style of Tai Chi (Yang, Chen, Sun, etc.) was used in the intervention described?	23 (74)
(6) What were the major components (i.e. slow movements, mental concentration and deep breathing) of Tai Chi that were emphasized in the training?	17 (55)
(7)^b^Were specific Tai Chi movements used in the training described and illustrated?	6 (19)
(8) What were the credentials of the Tai Chi instructors in the study?	12 (39)
(9) Was the evaluation of the Tai Chi training and/or the instructor by study subjects available?	2 (6)
(10) Was the description of the control comparable to the description of the Tai Chi training?	26 (84)

^a^Number of studies that satisfied reporting criterion.

^b^If well-established Tai Chi forms (i.e., the 24-forms of simplified Yang style Tai Chi [13] and the 10 forms described by Wolf et al. [14] are taught and proper references are provided, item 7 is considered satisfied.

**Table 3:** List of clinical applications that Tai Chi has been tested for in RCTs published between 1992 and 2007.

Clinical application	Number of trials (Reference numbers)
Balance improvement and fall prevention	16 [16, 17, 20, 21, 25, 29, 32, 36, 39, 40, 43, 48, 50, 53–55]
Osteoarthritis	4 [23, 27, 46, 47]
Quality of life among patients with chronic disease	3 [33, 35, 42]
Psychological health	3 [15, 18, 44]
Bone health	2 [30, 51]
Hypertension	2 [22, 28]
Immune health	2 [26, 49]
Heart failure	2 [34, 45]
Physical functions	1 [24]
Tension-type headaches	1 [56]
Sleep problems	1 [31]
Reduction in cardiovascular disease risk factors	1 [37]
Traumatic brain injury	1 [41]
Diabetes control	1 [52]
Rehabilitation following acute myocardial infarction	1 [19]
General fitness	1 [38]

**Table 4:** Distribution of Tai Chi clinical trials according to the number of items reported in the mini-checklist for specifying Tai Chi interventions.

Number of criteria reported	Number of articles (%)
10	—
9	—
8	—
7	7 (23)
6	11 (35)
5	9 (29)
4	3 (10)
3	1 (3)
2	—
1	—
